# A Ku-Band Fully Differential Low-Power High-Input P1dB Low-Noise Amplifier

**DOI:** 10.3390/nano14231913

**Published:** 2024-11-28

**Authors:** Sang-Rok Lee, Joon-Hyung Kim, Min-Seok Baek, Choul-Young Kim

**Affiliations:** 1 Department of Electronics Engineering, Chungnam National University, Daejeon 34134, Republic of Korea; sangrok.lee.85@gmail.com (S.-R.L.);; 2 Korea Aerospace Research Institute, Daejeon 34133, Republic of Korea

**Keywords:** Ku-band, fully differential, low-noise amplifier, input P1dB, class AB, CMOS, low power

## Abstract

This paper introduces a Ku-band fully differential low-power high-input 1 dB compression point (P1dB) low-noise amplifier (LNA). A fully differential structure is employed to enhance the input P1dB, common-mode noise rejection, and second harmonic cancellation. The first stage adopts large transistors and is optimized for power consumption and noise figure (NF). The output stage is designed with class AB bias, resulting in improved P1dB, power consumption, and linearity. The proposed two-stage fully differential common-source (CS) LNA was implemented using 65 nm bulk complementary metal oxide semiconductor (CMOS) technology. The fabricated LNA achieved a minimum NF of 2.7 dB at 13.6 GHz. Furthermore, it achieved a maximum gain of 19.92 dB at 12.2 GHz. Additionally, the LNA has an input P1dB of −7.45 dBm and an output power 1 dB compression point (OP1dB) of 10.09 dBm, both measured at 15.6 GHz. The LNA operates with a power consumption of 11 mW at a 1 V supply, and occupies a core size of 0.75 mm × 0.35 mm.

## 1. Introduction

In modern society, there is an increasing demand for advanced wireless communication for the rapid delivery of large amounts of data. In this respect, 5G communication using the FR2 band has an advantage in data transmission speed because the carrier frequency and bandwidth are higher than the FR1 band and other communication methods. However, the 5G FR2 band has low cell coverage due to its high attenuation characteristics. These characteristics act as an economic burden for the establishment of communication infrastructure such as base stations and repeaters, challenging commercialization. Therefore, in 5G-Advanced and 6G, the FR3 band is expected to emerge as a realistic candidate for near-future deployment. Compared to the FR2 band, the FR3 band has an appropriate transmission speed, relatively good attenuation characteristics, and improved cell coverage [1,2,3]. Factors such as low cost, low power consumption, integration capability, and mass production are required to build a wireless infrastructure covering a large area [4]. CMOS technology is recognized as a core technology that can be used to produce RFICs for wireless communication [5]. In this paper, we introduce a low-noise amplifier (LNA) design that operates in the Ku-band within the FR3 band using bulk CMOS technology. The signal received by the antenna is amplified by the first gain stage, the low-noise amplifier, with a minimal increase in the noise figure. In state-of-the-art communication systems, a base station transmits significant power-level signals, taking into account dense urban area and high-frequency attenuation characteristics [6]. As a result, an LNA with a low input P1dB is prone to saturation. Consequently, enhancing techniques for input P1dB are required when dealing with signals with such large dynamic ranges. This allows for the prevention of signal distortion, thereby expanding the linear operating range of the LNA. Additionally, low power consumption is also essential. However, the previously designed LNA includes a single-ended stage, which shows limitations when handling high dynamic range signals and is vulnerable to common-mode noise [7,8,9,10,11,12]. Therefore, we aim to introduce a fully differential LNA structure that enhances stability against common-mode noise and improves the input P1dB [13]. The differential structure effectively leverages the increased total width of the transistor compared to the single-ended structure, which is advantageous for power handling. Moreover, they exhibit better ground plane quality, lower parasitic effects of chip interconnections, and easier coupling between stages [14]. The proposed two-stage low-noise amplifier is a fully differential common-source (CS) structure with a gain of 19.92 dB. A large transistor technique was employed to secure a low noise figure. A large transistor is implemented by connecting multi-finger transistors in parallel [15]. At the same time, capacitive neutralization utilizing the advantages of differential structures was applied. This method ensures sufficient gain while maintaining stability. Along with this, class AB bias is applied to the output stage, allowing for sufficient P1dB and low-power characteristics simultaneously. The remainder of this article is organized as follows: Section 2 covers the detailed circuit design of the proposed LNA. Section 3 presents the measurement results and a comparative analysis of performance with the latest LNAs. Lastly, Section 4 concludes this paper by summarizing the key findings and contributions of this research.

## 2. Circuit Design

**Figure 1:**
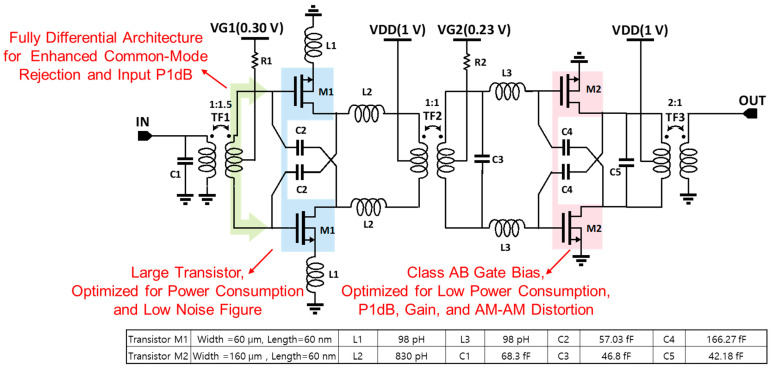
Schematic of the proposed low-noise amplifier.

**Figure 2:**
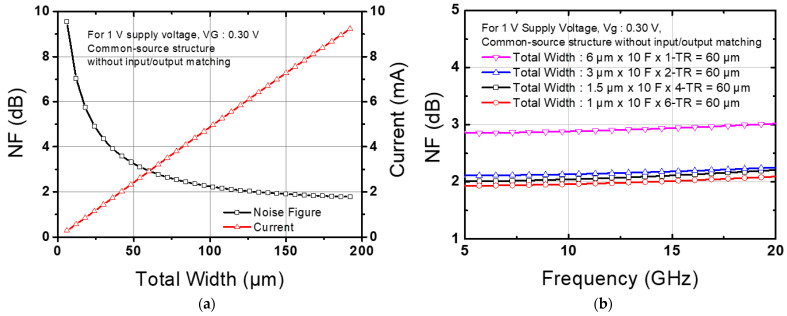
Simulated noise figure versus (**a**) transistor width and (**b**) number of transistors connected in parallel for same device size.

This paper proposes a design for a low-noise amplifier with enhanced common-mode noise rejection properties, high input P1dB, and low-power-consumption characteristics. Additionally, as detailed in reference [16], the differential architecture is advantageous for reducing harmonic-induced distortion due to the cancellation of the second harmonic component at the balun and virtual ground node. Figure 1 depicts the schematic of the proposed amplifier. A fully differential two-stage CS–CS structure has been selected to improve handling capability for high dynamic range signals under a low supply voltage of 1 V while ensuring adequate gain. This configuration offers several advantages. By introducing a differential architecture, it facilitates a higher input P1dB compared to a single-ended counterpart. Additionally, it allows for the application of neutralizing capacitors and is resistant to common-mode noise. Introducing a differential structure instead of a single-ended structure may double power consumption due to the increased total width of the transistors and can also deteriorate the noise figure because of additional components. To address this issue, we applied large transistors to the CS structure. Compared to a cascode structure, the CS structure offers advantages in minimizing power consumption by operating at lower supply voltages. Evaluating the trend of change in the noise figure while increasing the width of the transistor contributes to an improvement in the noise figure. Moreover, increasing the number of parallel transistors while maintaining a constant total width configuration offers the advantage of reducing total gate resistance, thus improving the noise figure [17]. To find the optimal size for the M1 transistor in the first stage, which has the most significant impact on the noise figure, schematic-level simulations were performed using a process design kit. Figure 2a illustrates the noise figure and power consumption with respect to the transistor width variations ranging from 6 to 192 μm at 14 GHz. Power consumption increases linearly as the width of the transistor increases. However, the noise figure decreases nonlinearly. Although noise figure improvement at the low-current region is large, it is difficult to select a transistor with a small width due to the large absolute noise figure value. Furthermore, when the width of the transistor is wider than 60 μm, a current increase of more than two times is required to achieve a 20% improvement in the noise figure. Therefore, considering this tendency, the width of the M1 transistor was selected as 60 μm. Figure 2b demonstrates an improvement in the noise figure as the total width of the transistor is fixed at 60 μm and the number of transistors connected in parallel (multipliers) is increased. The enhancement in the noise figure due to the increase in multipliers is clearly evident, with the improvement being pronounced up to six multipliers. As a result, six transistors with a gate width of 1 μm were selected and used in parallel. Additionally, we applied a source degeneration inductor L1 to facilitate matching between the optimum noise impedance and input impedance [18].

**Figure 3:**
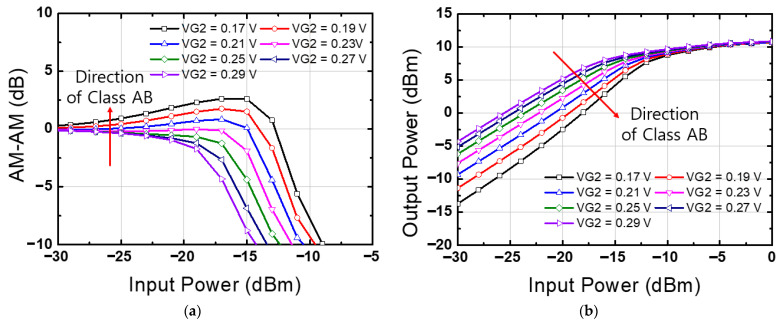
Simulation results of the proposed low-noise amplifier according to the second-stage bias (VG2) condition at 14 GHz. (**a**) AM-AM, (**b**) input power versus output power.

The bias setting for the output stage, where final amplification is achieved, involves a trade-off among several factors. These factors include power consumption, gain, distortion, and the maximum signal handling level of the amplifier. In general, class A biasing, commonly used for an LNA, provides high gain but makes it challenging to optimize power consumption and limits the handling of a high-power-level input signal. Therefore, applying class AB bias to the output stage of a low-noise amplifier can be a good choice for achieving high power efficiency and a high P1dB [19,20]. Typically, class AB biasing can result in reduced gain and increased signal distortion compared to class A. Therefore, in this paper, we carefully varied the bias and conducted thorough simulations. Figure 3 represents input power versus output power characteristics and amplitude-to-amplitude (AM-AM) distortion levels with respect to bias variation at 14 GHz. Considering changes in gain according to class variation, as well as P1dB and AM-AM distortion, a 0.23 V class AB level was chosen.

To ensure stability while maintaining gain performance, neutralization capacitors (C2 and C4) were adopted [21,22,23]. Additionally, inter-stage gain was boosted by introducing L2 at the output of M1 and L3 at the input of M2 [7,17]. To achieve input, inter-stage, and output matching, three transformers (TF1, TF2, and TF3) along with shunt capacitors (C1, C3, and C5) were utilized. A top metal layer with a thickness of 3.4 μm was used to construct all transformers, which is the thickest metal layer with low resistance and small silicon substrate-related parasitics. The turns ratios were 1:1.5, 1:1, and 2:1. Their metal width was designed to be 3 μm and the metal spacing was 2 μm. Electromagnetic simulation was performed to check the coupling coefficient of the transformers. The average values of 0.674 for TF1, 0.686 for TF2, and 0.68 for TF3 were confirmed.

## 3. Measurement

**Figure 4:**
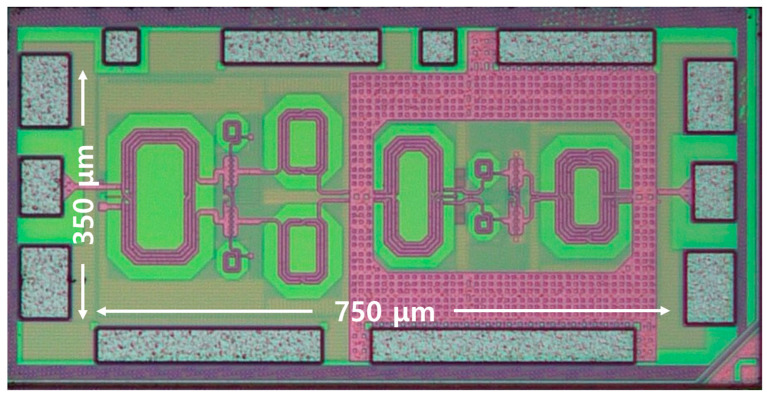
Microphotograph of the proposed fully differential low-noise amplifier.

**Figure 5:**
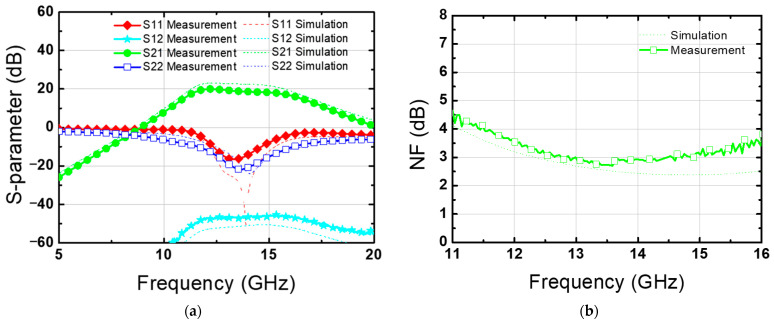

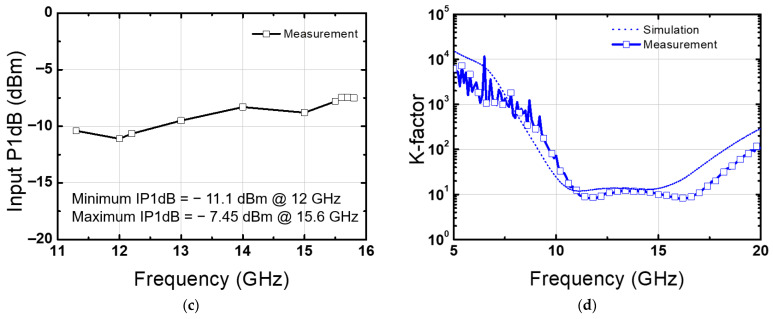
(**a**) Simulated and measured S-parameter, (**b**) simulated and measured noise figure, (**c**) measured input P1dB, (**d**) simulated and measured K-factor (stability).

**Figure 6:**
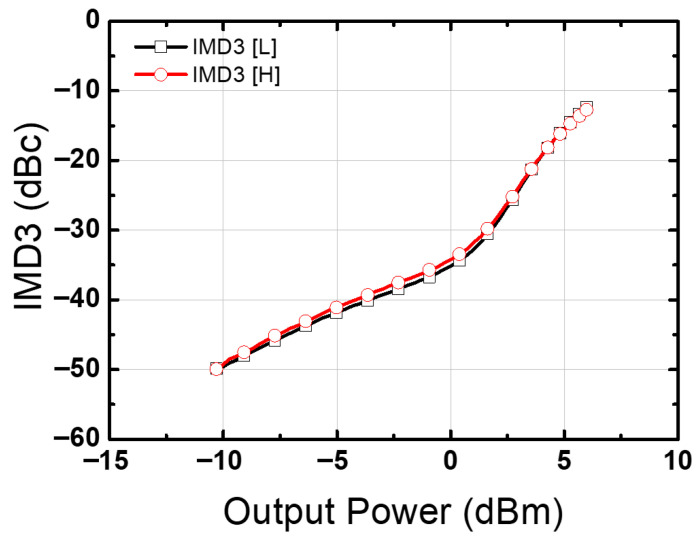
Measured third-order intermodulation distortion (IMD3) at 14 GHz (100 MHz tone spacing).

Figure 4 shows a microphotograph of a chip fabricated with 65 nm bulk CMOS technology. The size of the chip was 0.75 mm × 0.35 mm (0.26 mm^2^). An on-chip GSG probe was used with a network analyzer for S-parameter measurements. The operating voltage of the chip was 1 V, and it consumed 11 mW of static power. Additionally, the power consumption measured at the OP1dB point at 14 GHz was 30 mW. Figure 5 presents the S-parameter, noise figure, input P1dB, and K-factor (stability) measurement results. The maximum gain was 19.92 dB at 12.2 GHz with a −3 dB bandwidth of 4.65 GHz (11.24–15.89 GHz). The noise figure reached its minimum value of 2.7 dB at 13.6 GHz. Additionally, at a frequency of 15.6 GHz, the input P1dB was −7.45 dBm and the output P1dB was 10.09 dBm. Figure 6 shows the third-order intermodulation distortion (IMD3) measured using tones with an interval of 100 MHz at 14 GHz. It shows a low IMD3 value of less than −30 dBc, even at output powers above 0 dBm or higher. Table 1 compares the measurement results of the proposed LNA with those of the latest Ku-band LNA, which utilizes a process comparable to the 65 nm CMOS process used in the proposed LNA. For a fair comparison, two figure of merits were considered [15]. Utilizing comparison tables and figure of merit (FoM) enable a fair assessment of the performance of the LNA. From the comparison table, it can be confirmed that the proposed LNA exhibits the best input P1dB performance. Regarding figure of merit I, it demonstrates adequate performance. However, regarding figure of merit II, the proposed LNA shows the best performance among the cited literature.

(1) $$\mathrm{FoM}\;I=\frac{\mathrm{Gain}\left[\mathrm{abs}.\right]}{\left(\mathrm{Noise}\;\mathrm{Factor}-1\right)\left[\mathrm{abs}.\right]\cdot P_{\mathrm{DC}}\left[\mathrm{mW}\right]}$$

(2) $$\mathrm{FoM}\;\mathrm{II}=\frac{\mathrm{Gain}\left[\mathrm{abs}.\right]\cdot \mathrm{Input}\;P1\mathrm{dB}\left[\mathrm{mW}\right]}{\left(\mathrm{Noise}\;\mathrm{Factor}-1\right)\left[\mathrm{abs}.\right]\cdot P_{\mathrm{DC}}\left[\mathrm{mW}\right]}\;$$

## 4. Conclusions

In this paper, we designed and implemented a low-power and high-input P1dB low-noise amplifier operating in the Ku-band. The proposed LNA achieved a high input P1dB and a low noise figure by adopting a fully differential structure and large transistor. Additionally, by applying class AB bias at the output stage, we optimized power consumption while securing a high P1dB. In addition, the fully symmetric differential structure improves the rejection of common-mode noise. The results of this paper demonstrate the best input P1dB performance compared to the latest Ku-band LNAs.

## Figures and Tables

**Table 1:** Performance summary and comparison with state-of-the-art Ku-band LNAs.

Ref.	This Work	[7]	[8]	[9]	[10]	[11]	[12]
Technology	65 nm CMOS	65 nm CMOS	40 nm CMOS	65 nm CMOS	65 nm CMOS	65 nm CMOS	130-nm SiGe
Supply Voltage [V]	1	0.8	1	1.2	1	1	1
Topology	Diff. (CS)-Diff. (CS)	Single (CS)-Single (CS)	Single (Cascode)	Single (CS)-Single (CC)	Single (CG)-Single (CS)	Single (CC)-Diff. (CS)	Single (CE)-Single (CE)
Frequency (GHz)	11.24–15.89	6.7–15.3	10–14	7.2–27.3	7.6–29	9.2–12.7	10–22
3 dB-BW(GHz)	4.65	8.6	4	20.1	21.4	3.5	12
Peak Gain (dB)	19.92 @ 12.2 GHz	20	11	16.6	10.7	19.5	15.5
Noise Figure Minimum (dB)	2.7 @ 13.6 GHz	2.08 (Average)	1.7	3.3	4.5	2.3	3.2
Input P1dB (dBm)	−7.45 @ 15.6 GHz	−17	−8.8	−11.7	NA	−13.5	NA
P_DC_ (mW)	11	12.8	10	13.2	12.1	5.9	4
Core Area (mm^2^)	0.26	0.144	0.162	0.14	0.3	0.8	0.1
FoM I	10.35	12.72	2.63	3.04	0.53	21.64	8.14
FoM II	1.86	0.25	0.35	0.21	NA	0.97	NA

BW: bandwidth, Diff.: differential, Single: single-ended, CS: common source, CC: cascode, CE: common emitter, FoM: figure of merit.

## Data Availability

The original contributions presented in the study are included in the article.
